# RIP1 protects melanoma cells from apoptosis induced by BRAF/MEK inhibitors

**DOI:** 10.1038/s41419-018-0714-7

**Published:** 2018-06-07

**Authors:** Fu Xi Lei, Lei Jin, Xiao Ying Liu, Fritz Lai, Xu Guang Yan, Margaret Farrelly, Su Tang Guo, Xin Han Zhao, Xu Dong Zhang

**Affiliations:** 1 Department of Medical Oncology, The First Affiliated Hospital of Xi’an Jiaotong University, Xi’an, Shaanxi 710061 China; 2 School of Medicine and Public Health, The University of Newcastle, Newcastle, NSW 2308 Australia; 3 School of Life Science, Anhui Medical University, Hefei, Anhui 230032 China; 4 Department of Molecular Biology, Shanxi Cancer Hospital and Institute, Taiyuan, Shanxi 030013 China

## Abstract

Many recent studies have uncovered the necessary role for the receptor-interacting protein kinase 1 (RIP1) in regulating apoptosis and necrosis that cells undergo in response to various cellular stresses. However, the functional significance of RIP1 in promoting cancer cell survival remains poorly understood. Here, we report that RIP1 was upregulated and contributed to both intrinsic and acquired resistance of melanoma cells to BRAF/MEK inhibitors through activation of NF-κB. Strikingly, Snail1-mediated suppression of CYLD played a crucial role in promoting RIP1 expression upon ERK activation, particularly, in melanoma cells with acquired resistance to BRAF inhibitors. In addition, RIP1 kinase activity was not required for melanoma cells to survive BRAF/MEK inhibition as RIP1 mediated NF-κB activation through its intermediate domain. Collectively, our findings reveal that targeting RIP1 in combination with BRAF/MEK inhibitors is a potential approach in the treatment of the disease.

## Introduction

Targeting BRAF, MEK, and co-targeting BRAF and MEK using specific inhibitors have become the standard of care for patients with late-stage mutant BRAF melanomas^1^. However, the therapeutic benefits are often of limited duration due to rapid development of resistance^2^. Among many mechanisms that are involved in resistance of melanoma to BRAF/MEK inhibitors is reactivation of the RAF/MEK/ERK pathway, which is found in ~80% of melanomas with acquired resistance to BRAF/MEK inhibitors^3^. A number of mechanisms have been shown to contribute to reactivation of the pathway, such as the expression of BRAF splice variants^4^, BRAF amplification^5^, secondary active mutations in NRAS or MEK1/2^6^, signaling switching to CRAF^2^, and increased expression of MAP3K8 (COT)^7^.

Although blockade of the RAF/MEK/ERK pathway has been well demonstrated to inhibit melanoma cell proliferation, induction of apoptotic cell death has also been shown in varying in vitro and in vivo models^8^. Regression of metastatic BRAF melanomas is a common response to administration of BRAF/MEK inhibitors in patients^9^, suggesting that apoptosis induction may be a major biological consequence of inhibition of the pathway that causes remission of melanomas^10^. In support of this notion, we have previously shown that induction of apoptosis is a major determinant of long-term responses of BRAF^V600E^ melanoma cells to mutant BRAF inhibitors^9^. Nevertheless, molecular mechanisms responsible for resistance of melanoma cells to apoptosis induced by inhibition of the pathway remain to be fully understood.

Receptor-interacting protein kinase 1 (RIP1) is a protein Ser/Thr kinase that mediates both cell survival and death signaling and is an important determinant of cell fate in response to cellular stress, in particular, to activation of death receptors such as TNF receptor 1 (TNFR1)^11, 12^. Upon TNFR1 stimulation, RIP1, along with other proteins including TRADD, TRAF2, cIAP1, and cIAP2, are recruited to form prosurvival complex I^13^. This results in stabilization of RIP1 through K63-linked polyubiquitination carried out by TRAF2/cIAPs^14^. Structurally, RIP1 comprised an N-terminal kinase domain, an intermediate domain and a carboxyl-terminal death domain^15^. Of note, the intermediate domain is critical for K-63-linked ubiquitination of RIP1, which binds to TAB2/TAB3/TAK1 complex and NEMO, thus leading to activation of NF-κB, which plays an important role in regulating many cellular processes such as cell survival and proliferation^16^. When K63-polyubiquitinated RIP1 is deubiquitinated by the deubiquitinase cylindromatosis (CYLD), RIP1 functions to promote apoptosis in cells with sufficient caspase-8 activation^17^. However, when caspase-8 activation is limited, deubiquitinated RIP1 recruits RIPK3 causing programmed necrosis (necroptosis) in some types of cells^13, 14, 17^.

The role of RIP1 in activation of NF-κB appears to be highly cell type-dependent. While RIP1 is not essential for TNFR1-induced canonical NF-κB activation in mouse embryonic fibroblasts^18^, we have previously demonstrated that RIP1 promotes the pathogenesis of human melanoma through activation of NF-κB^13^. Moreover, RIP1 plays an important role in protection of melanoma cells from apoptosis induced by endoplasmic reticulum (ER) stress^19^. In this report, we show that RIP1 protects human melanoma cells from apoptosis induced by BRAF/MEK inhibitors, and that this is mediated by activation of NF-κB. Moreover, we demonstrate that suppression of CYLD by ERK1/2 signaling plays an important role in maintaining RIP1 expression, and that melanoma cells with acquired resistance to BRAF inhibitors are more critically dependent on RIP1 for survival.

## Results

### RIP1 contributes to intrinsic resistance of melanoma cells to apoptosis induced by BARF/MEK inhibitors

**Fig. 1:**
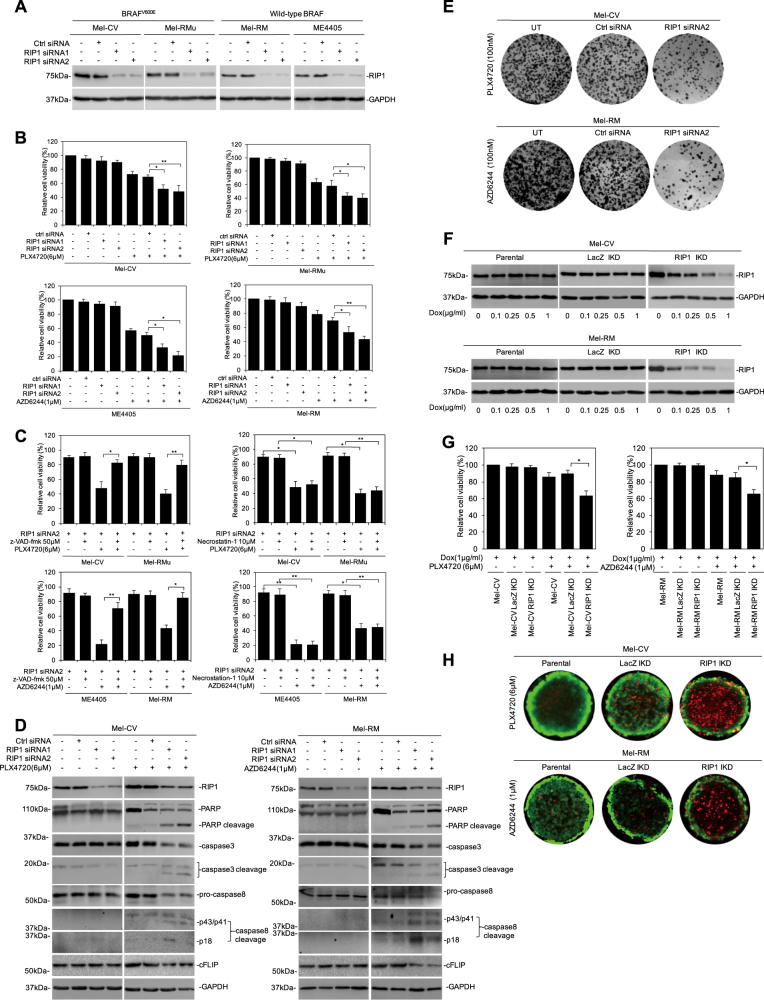
RIPK1 contributes to intrinsic resistance of melanoma cells to apoptosis induced by BARF/MEK inhibitors. **a** Mel-CV, Mel-RMu (BRAF^V600E^ mutant) and ME4405, Mel-RM (BRAF wild-type) were individually transfected with control siRNA or RIP1 siRNA. Twenty-four hours after transfection, whole-cell lysates were subjected to western blotting. *n* = 3. **b**–**e** Melanoma cells treated with PLX4720 (6 μM) or AZD6244 (1 μM) in the presence or absence of z-VAD-fmk (50 μM) or Necrostatin-1 (10 μM) for 72 h were subjected to Cell Titer-Glo assays (**b**, **c**), western blotting (**d**), and Clonogenic assay (**e**). Data are mean ± SE, *n* = 3. *P < 0.05, **P < 0.01, Student’s *t* test. **f** Whole-cell lysates from LacZ inducible knock down (LacZ IKD) and RIP1 inducible knock down (RIP1 IKD) melanoma cells treated with Doxycycline (1 μg/ml) for 72 h were subjected to western blotting. *n* = 3. **g** LacZ IKD and RIP1 IKD melanoma cells treated with Doxycycline (1 μg/ml) in combination with PLX4720 (6 μM) or AZD6244 (1 μM) for 72 h were subjected to cell Titer-Glo assays. Data are mean ± SE, *n* = 3. *P < 0.05, Student’s *t* test. **h** LacZ IKD and RIP1 IKD melanoma cells grown in three-dimensional culture treated with Doxycycline (1 μg/ml) in combination with PLX4720 (6 μM) or AZD6244 (1 μM) for 7–10 d were stained with calcein AM (living cell stain) and ethidium homodimer-1 (dead cell stain) for 30 min followed by observation under microscope. Data are mean ± SE, *n* = 3. *P < 0.05, Student’s *t* test

To examine the potential effect of RIP1 on the response of melanoma cells to treatment with BRAF/MEK inhibitors, we silenced RIP1 using two individual siRNAs in two BRAF^V600E^ melanoma cell lines (Mel-CV and Mel-RMu) and two wild-type BRAF melanoma cell lines (ME4405 and Mel-RM). Consistent with previous results, RIP1 silencing alone did not trigger cell death (Fig. 1a, b)^13^. However, it caused further reduction in cell viability in Mel-CV and Mel-RMu cells treated with the BRAF inhibitor PLX4720 and in ME4405 and Mel-RM cells treated with the MEK inhibitor AZD6244 (selumetinib) (Fig. 1a, b). This was associated with activation of caspase-8 and caspase-3, cleavage of PARP and decreases in cFLIP expression levels (Fig. 1d). Moreover, treatment with the general caspase inhibitor z-VAD-fmk abolished reduction in cell viability (Fig. 1c). These results indicate that RIP1 plays a role in intrinsic resistance of melanoma cells to apoptosis induced by BRAF/MEK inhibitors. In accordance, silencing of RIP1 further reduced clonogenecity in Mel-CV cells treated with PLX4720 and in Mel-RM cells treated with AZD6244 (Fig. 1e). Of note, treatment with necrostatin-1, a specific inhibitor of RIP1 kinase activity, did not affect apoptosis of Mel-CV and Mel-RM cells treated respectively with PLX4720 and AZD6244 (Fig. 1c), suggesting that RIP1 kinase activity is not involved in protection of melanoma cells from apoptosis induced by BRAF/MEK inhibitors.

To facilitate further investigation, we established Mel-CV and Mel-RM sublines carrying a system with inducible RIP1 shRNA in response to doxycycline (DOX) (Fig. 1f). Induced silencing of RIP1 similarly enhanced apoptosis in Mel-CV and Mel-RM cells triggered by PLX4720 and AZD6244, respectively (Fig. 1g). Moreover, it promoted killing of Mel-CV cells by PLX4720 and Mel-RM cells by AZD6244 grown in 3-dimensional (3D) cultures (Fig. 1h).

### RIP1 is upregulated in melanoma cells with acquired resistance to PLX4720

**Fig. 2:**
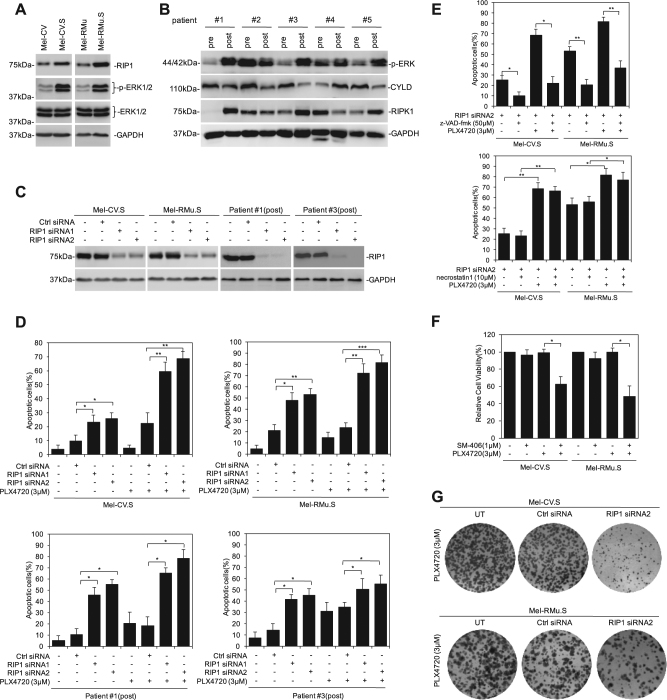
RIPK1 is upregulated in melanoma cells with acquired resistance to PLX4720. **a** Whole-cell lysates of melanoma cells with acquired resistance to PLX4720 and their parental counterparts were subjected to western blotting. **b** Whole-cell lysates of fresh melanoma isolates of paired sets of pre-treatment (pre) and post-relapsed (post) patient specimens from five patients with relapsed BRAF^V600E^-expressing melanomas were subjected to western blotting. **c** Melanoma cells were individually transfected with either control siRNA or RIP1 siRNA. Twenty-four hours after transfection, whole-cell lysates were subjected to western blotting. **d**, **e** Melanoma cells treated with PLX4720 (3 μM) in the presence or absence of z-VAD-fmk (50 μM) or Necrostatin-1 (10 μM) for 72 h were subjected to the apoptosis assay with Annexin V-FITC and PI. Data are mean ± SE, *n* = 3. *P < 0.05, **P < 0.01, ***P < 0.001, Student’s *t* test. **f** Melanoma cells treated with SM-406 (1 μM) in the presence or absence of PLX4720 (3 μM) for 72 h were subjected to Cell Titer-Glo assays. Data are mean ± SE, *n* = 3. *P < 0.05, Student’s *t* test. **g** Melanoma cells were individually transfected with either control siRNA or RIP1 siRNA. Cells were subjected to Clonogenic assay

To test whether RIP1 is also involved in acquired resistance of melanoma cells to BARF inhibitors, we examined its expression in Mel-CV and Mel-RMu cells selected for resistance to PLX4720 by prolonged exposure to the inhibitor (Mel-CV.S and Mel-RMu.S cells) in comparison with their parental counterparts^9^. In addition, we compared the expression of RIP1 between paired fresh melanoma isolates from patients before and after treatment with the BRAF inhibitor vemurafenib^20^. The levels of RIP1 appeared to be upregulated in Mel-CV.S and Mel-RMu.S compared with Mel-CV and Mel-RMu cells, respectively (Fig. 2a). Moreover, RIP1 expression was increased in three out of five fresh melanoma isolates from patients after treatment with vemurafenib (patients #1, 3, and 5) compared with paired pre-treatment samples (Fig. 2b). The increased expression of RIP1 was important for survival of melanoma cells that acquired resistance to BRAF inhibitors, in that RIP1 silencing by siRNA along caused reduction in cell viability in Mel-CV.S and Mel-RMu.S cells and in post-treatment fresh melanoma isolates, and re-sensitized these cells to PLX4720-induced cell death (Fig. 2c, d). These results suggest that melanoma cells with acquired resistance to BRAF inhibitors are more critically dependent on RIP1 for survival. Treatment with z-VAD-fmk, but not necrostatin-1, abolished the effect of RIP1 silencing on cell viability in the presence or absence of PLX4720 (Fig. 2e), indicating that RIP1 protects these cells from apoptosis, and that the kinase activity of RIP1 is not involved in the process. In accordance, the addition of the Smac mimetic SM-406 that is known to cause proteasomal degradation of cIAPs, thus resulting in reduciton in RIP1 expression, similarly rendered Mel-CV.S and Mel-RMu.S cells susceptible to PLX4720 (Fig. 2f). The protective role of RIP1 against PLX4720-induced apoptosis in Mel-CV.S and Mel-RMu.S cells was also reflected by reduced clonogenicity when RIP1 was silenced (Fig. 2g).

### RIP1 protects melanoma cells from PLX4720 through NF-κB

**Fig. 3:**
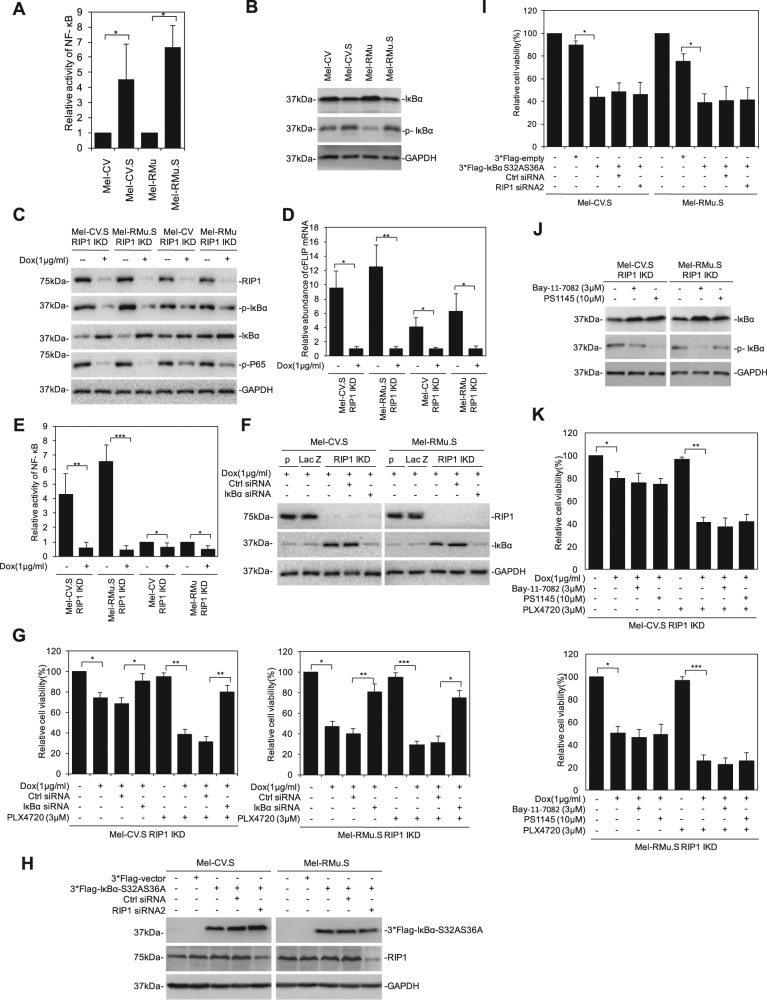
RIPK1 protects melanoma cells from PLX4720 through NF-κB. **a** Melanoma cells were transfected with the indicated reporter constructs and Renilla luciferase plasmid. Forty-eight hours after transfection, NF-κB transcriptional activity was determined by luciferase assays. Data are mean ± SE, *n* = 3. *P < 0.05, Student’s *t* test. **b** Whole-cell lysates of melanoma cells were subjected to western blotting. **c**–**e** Cells were treated with or without Doxycycline (1 μg/ml) for 72 h, whole-cell lysates were subjected to western blotting (**c**), total RNA from cells was subjected to qPCR analysis (**d**), cells were determined by NF-κB transcriptional activity by luciferase assays (**e**). Data are mean ± SE, *n* = 3. *P < 0.05, **P < 0.01, ***P < 0.001, Student’s *t* test. **f**, **g** Cells were individually transfected with either control siRNA or IκBα siRNA in presence of Doxycycline (1 μg/ml). Whole-cell lysates were subjected to western blotting (**f**). Cells were subjected to Cell Titer-Glo assyas (**g**). Data are mean ± SE, *n* = 3. *P < 0.05, **P < 0.01, ***P < 0.001, Student’s *t* test. **h**, **i** Cells individually transfected with either 3*Flag vector or 3*Flag-IκBα-S32AS36A vector were co-transfected with control siRNA or RIP1 siRNA. Whole-cell lysates were subjected to western blotting (**h**). Cells were subjected to Cell Titer-Glo assyas (**i**). Data are mean ± SE, *n* = 3. *P < 0.05, student’s *t* test. **j**, **k** Cells were treated with Bay-11-7082 (3 μM) or PS1145 (10 μM) in presence of Dox (1 μg/ml), respectively. Forty-eight hours after treatment, whole-cell lysates were subjected to western blotting (**j**), cells were subjected to Cell Titer-Glo assyas (**k**). Data are mean ± SE, *n* = 3. *P < 0.05, **P < 0.01, ***P < 0.001, Student’s *t* test

In agreement with previous results, constitutive activation of NF-κB was readily detectable in melanoma cells (Fig. 3a). Of note, it was markedly increased in Mel-CV.S and Mel-RMu.S cells compared with their corresponding parental counterparts (Fig. 3a)^20^. The increased activation of NF-κB in PLX4720-selected cells was confirmed by decreased expression of inhibitor of NF-κB α (IκBα) and increased phosphorylation of the protein in the PLX4720-selected cells (Fig. 3b)^9^. Silencing of RIP1 significantly caused the reduction in phosphorylated IκBα, phosphorylated p65, the mRNA expression level of cFLIP and the relative activity of NF-κB in Mel-CV.S and Mel-RMu.S and, to a less extent, in their parental counterparts Mel-CV and Mel-RMu (Fig. 3c–e), indicating that the increased expression of RIP1 plays a role in NF-κB activation. Furthermore, the combination of a TNFα blocking antibody and PLX4720 further reduced the cell viability of Mel-CV.S and Mel-CV, suggesting that autocrine TNFα driven by RIP1-NF-κB signaling as shown previously may contribute to resistance of melanoma cells to BRAF inhibitors (Fig. S1)^13^. In contrast, there was no significant difference in activation (phosphorylation) of p38 and JNK between melanoma cells resistant to PLX4720 and their parental counterparts (Fig. S2a). Moreover, RIP1 knockdown did not affect P38 and JNK activation (Fig. S2b). The functional significance of NF-κB in RIP1-mediated protection of melanoma cells from PLX4720 was demonstrated by silencing of IκBα with siRNA, which caused hyperactivation of NF-κB and abolished killing of Mel-CV.S and Mel-RMu.S cells by RIP1 silencing with or without the addition of PLX4720 (Fig. 3f, g)^20^. Moreover, IκBα silencing diminished killing by PLX4720 in Mel-CV with or without RIP1 silenced (Fig. S3)^13^. RIP1 silencing did not cause any further reduction in the viability of Mel-CV.S and Mel-RMu.S cell transduced with a non-degradable mutant of IκBα (IκBαS32AS36A) (Fig. 3h, i), which diminished NF-κB activity^13^, confirming the role of NF-κB in RIP1-mediated protection of melanoma cells from killing by PLX4720. Similarly, the addition of the NF-κB inhibitor PS1145 or Bay-11-7082 induced no further killing in Mel-CV.S and Mel-RMu.S cell with RIP1 silenced in the absence or presence of PLX4720 (Fig. 3j, k).

### CYLD downregulation is responsible for upregulation of RIP1 in melanoma cells with acquired resistance to BRAF inhibitors

**Fig. 4:**
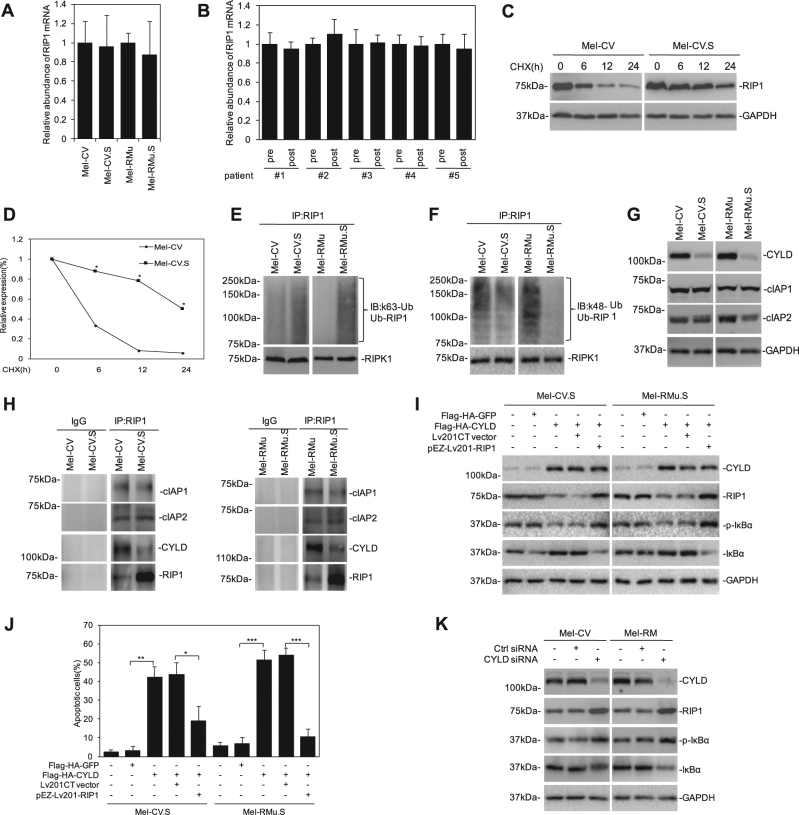
CYLD downregulation is responsible for upregulation of RIPK1 in melanoma cells with acquired resistance to BRAF inhibitors. **a**, **b** Total RNA from melanoma cells was subjected to qPCR analysis. Data are mean ± SE, *n* = 3. *P < 0.05, Student’s *t* test. **c** Whole-cell lysates from cells treated with cycloheximide (CHX; 1 μg/ml) for indicated periods of time were subjected to western blotting. **d** Quantitation of the western blot bands as shown in **c**. **e**, **f** RIP1 immunoprecipitates from cells treated with MG132 (5 μM) for 6 h were subjected to western blotting. **g** Whole-cell lysates were subjected to western blotting. **h** RIP1 immunoprecipitates from whole-cell lysates were subjected to western blotting. **i**, **j** Cells individually transfected with either Flag-HA-GFP or Flag-HA-CYLD were co-transfected with Lv201CT Vector or pEZ-Lv201-RIP1. Forty-eight hours after transfection, whole-cell lysates were subjected to western blotting (**i**), cells were subjected to the apoptosis assay with Annexin V-FITC and PI (**j**). Data are mean ± SE, *n* = 3. *P < 0.05, **P < 0.01, ***P < 0.001, Student’s *t* test. **k** Whole-cell lysates from cells transfected with either control siRNA or CYLD siRNA were subjected to western blotting

Having demonstrated the functional importance of RIP1 in protection of melanoma cells from apoptosis induced by BRAF/MEK inhibitors and the mechanism involved, we focused on investigation of the mechanism responsible for upregulation of RIP1 in melanoma cells with acquired resistance. As shown in Fig. 4a, there was no significant change in the expression levels of RIP1 mRNA between Mel-CV.S and Mel-RMu.S and their corresponding parental counterparts, nor was there any difference in RIP1 mRNA expression levels between fresh melanoma isolates from patients before and after treatment with vemurafenib (Fig. 4b), suggesting that upregulation of RIP1 in melanoma cells with acquired resistance was due to posttranscriptional regulation. Indeed, the turnover rate of the RIP1 protein was markedly slower in Mel-CV.S than Mel-CV cells (Fig. 4c, d). Moreover, K63-linked polyubiquitination of RIP1 was increased, whereas K48-linked polyubiquitination of RIP1 was reduced in Mel-CV.S and Mel-RMu.S cells in comparison with their corresponding parental counterparts (Fig. 4e, f). Therefore, upregulation of RIP1 in melanoma cells with acquired resistance to BRAF inhibitors is primarily due to increased stability of the protein.

Polyubiquitination of RIP1 is mediated by cIAPs, whereas the deubiquitinase CYLD is involved in deubiquitinating RIP1^21^. We therefore examined the expression levels of cIAPs and CYLD in Mel-CV.S and Mel-RMu.S in comparison with Mel-CV and Mel-RMu cells, respectively. Remarkably, while there was no difference in the expression of cIAP1 and cIAP2, the levels of CYLD were downregulated in Mel-CV.S and Mel-RMu.S cells (Fig. 4g). In accordance, the amount of CYLD co-precipitated with RIP1 was reduced in Mel-CV.S and Mel-RMu.S cells, whereas the amount of cIAP1 and cIAP2 co-precipitated with RIP1 remained largely unaltered (Fig. 4h). Overexpression of CYLD in Mel-CV.S and Mel-RMu.S cells triggered apoptosis, which was associated with reduction in RIP1 expression, the increase in IκBα and the decrease in its phosphorylation. This was reversed by co-overexpression of RIP1 (Fig. 4i, j), suggesting that downregulation of CYLD is responsible for upregulation of RIP1 in melanoma cells with acquired resistance to BRAF inhibitors. Moreover, Silencing of CYLD in Mel-CV and Mel-RM caused an increase in RIP1 and phosphorylated IκBα (Fig. 4k). In support, CYLD is reduced in post-treatment fresh melanoma isolates with increased levels of RIP1 compared with paired pre-treatment samples (Fig. 2b).

### ERK activation promotes RIP1 expression through Snail1-mediated suppression of CYLD

**Fig. 5:**
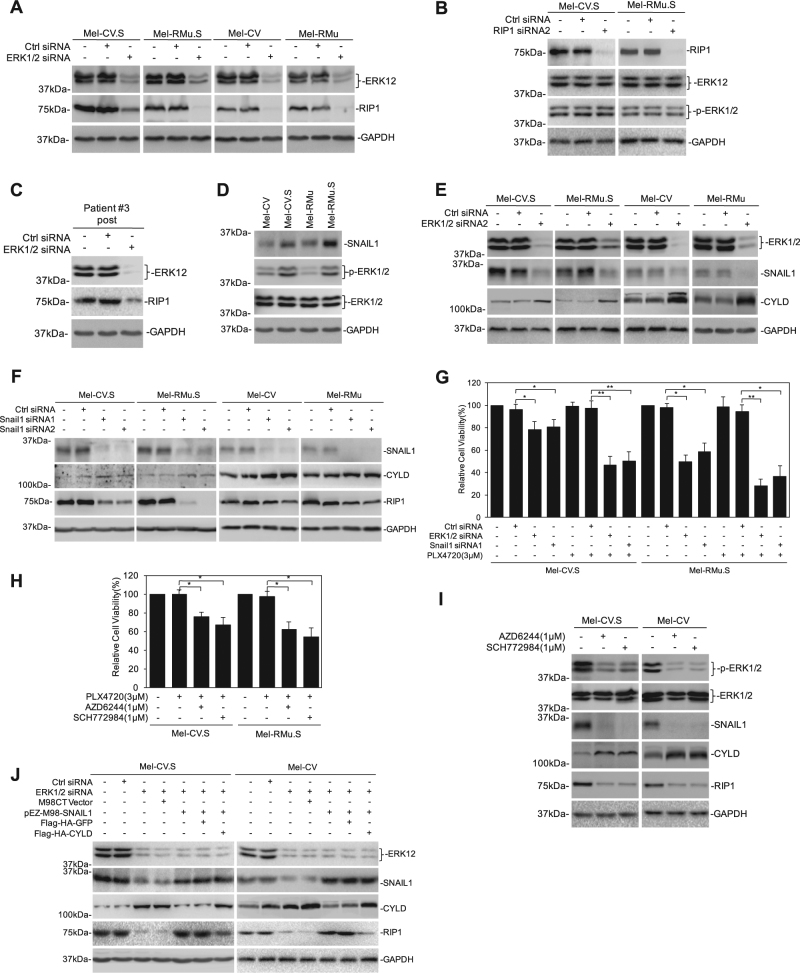
ERK activation promotes RIPK1 expression through Snail1-mediated suppression of CYLD. **a** Whole-cell lysates from melanoma cells individually transfected with either control siRNA or ERK1/2 siRNA were subjected to western blotting. **b** Whole-cell lysates from melanoma cells individually transfected with either control siRNA or RIP1 siRNA were subjected to western blotting. **c** Whole-cell lysates from melanoma cells individually transfected with either control siRNA or ERK1/2 siRNA were subjected to western blotting. **d** Whole-cell lysates from melanoma cells were subjected to western blotting. **e** Whole-cell lysates from cells individually transfected with either control siRNA or ERK1/2 siRNA were subjected to western blotting. **f** Whole-cell lysates from cells individually transfected with either control siRNA or Snail1 siRNA were subjected to western blotting. **g** Cells transfected with ERK1/2 siRNA or Snail1 siRNA in the presence or absence of PLX4720 (3 μM) for 72 h were subjected to Cell Titer-Glo assays. Data are mean ± SE, *n* = 3. *P < 0.05, **P < 0.01, Student’s *t* test. **h** Cells treated with combination of PLX4720 (3 μM) and AZD6244 (1 μM) or SCH772984 (1 μM) were subjected to Cell Titer-Glo assays. Data are mean ± SE, *n* = 3. *P < 0.05, Student’s *t* test. **i** Whole-cell lysates from cells treated with AZD6244 (1 μM) or SCH772984 (1 μM) were subjected to western blotting. **j** Cells transfected with ERK1/2 siRNA were co-transfected with pEZ-M98-SNAIL1 or Flag-HA-CYLD. Forty-eight hourswafter transfection, whole-cell lysates were subjected to western blotting

As reported before, ERK activation was increased in melanoma cells selected for resistance to PLX4720 (Fig. 2a)^9^. Similarly, phosphorylated ERK1/2 was elevated in post-treatment fresh melanoma isolates compared with paired pre-treatment samples, which was largely associated with the increase in RIP1 expression (Fig. 2b and S4). We therefore investigated whether there was a causal relationship between the increased activation of ERK and upregulation of RIP1 in Mel-CV.S and Mel-RMu.S cells. As shown in Fig. 5a, combined silencing of ERK1 and ERK2 resulted in reduction in RIP1 expression not only in Mel-CV.S and Mel-RMu.S cells, but also in Mel-CV and Mel-RMu cells, indicating that ERK1/2 signaling promotes RIP1 expression in melanoma cells. On the other hand, RIP1 silencing did not impinge on activation of ERK1/2 (Fig. 5b). Consistent with the role of ERK1/2 activation in RIP1 expression in melanoma cells, silencing of ERK1/2 similarly caused downregulation of RIP1 in fresh melanoma isolates (Fig. 5c).

Since the expression of CYLD is transcriptionally repressed by Snail1^22^, and downregulation of CYLD results in an increase in RIP1 in melanoma cells, we examined whether ERK1/2-mediated expression of RIP1 is related to suppression of CYLD by Snail1. Indeed, similar to activated ERK1/2 and RIP1, Snail1 was upregulated in Mel-CV.S and Mel-RMu.S compared with Mel-CV and Mel-RMu cells, respectively (Fig. 5d). Moreover, silencing of ERK1/2 caused downregulation of Snail1 and upregulation of CYLD, whereas silencing of Snail1 recapitulated the effect of ERK1/2 silencing on the expression of CYLD and RIP1 in Mel-CV.S and Mel-RMu.S cells, and to a lesser extent, in Mel-CV and Mel-RMu cells (Fig. 5e, f). In accordance, inhibition of ERK1/2 or Snail1 rendered Mel-CV.S and Mel-RMu.S cells sensitive to PLX4720 (Fig. 5g, h). Indeed, treatment with the MEK inhibitor AZD6244 or the ERK1/2 inhibitor SCH772984 decreased the expression of Snail1 and RIP1 and increased the expression of CYLD (Fig. 5i). These results suggest that promotion of Snail1 expression by ERK1/2 activation is important for ERK1/2-mediated upregulation of RIP1 in melanoma cells. In support, overexpression of Snail1 reduced CYLD expression and upregulated RIP1 in Mel-CV.S and Mel-CV cells even when ERK1/2 was silenced, whereas overexpression of CYLD diminished the effect of Snail1 overexpression on the expression of RIP1 (Fig. 5j).

## Discussion

Despite the diverse functions of RIP1 in regulation of cell survival and death signaling^11, 12^, many recent studies primarily focused on its role in mediating apoptotic and necrotic cell death induced by cellular stress^23, 24^. In particular, the involvement of RIP1 in apoptosis and necrosis of cancer cells in response to therapeutic drugs is being increasingly reported. Nevertheless, RIP1 knockout mice exhibit massive apoptosis in selected tissues and die at age 1–3 days after birth^12^, suggesting that RIP1 is important for cell survival conceivably in a cell type- and context-dependent manner. Indeed, while overexpression of RIP1 triggers cell death in many types of cells^12, 25^, we have recently shown that RIP1 is commonly upregulated and functions as an oncogenic driver in human melanoma, and that it promotes survival of melanoma cells undergoing ER stress^13, 19^. In accordance, we found in this study that the expression of RIP1 was an important mechanism that was involved in intrinsic resistance of melanoma cells to BRAF/MEK inhibitors and played a role in survival of melanoma cells with acquired resistance to BRAF inhibitors. These findings not only further highlight the biological importance of RIP1 in promoting survival of melanoma cells under cellular stress, but also bear important practical implications, in that intrinsic and acquired resistance of melanoma cells to inhibition of the RAF/MEK/ERK pathway remains a major barrier for curative treatment of the disease^4^.

RIP1-mediated resistance of melanoma cells to BRAF/MEK inhibitors was due to its inhibitory effect on induction of apoptosis, as RIP1 silencing promoted activation of the caspase cascade and a general caspase inhibitor abolished enhancement of killing of melanoma by silencing of RIP1. Moreover, protection of melanoma cells against apoptosis by RIP1 appeared to be mediated by NF-κB activity, in that hyperactivation of NF-κB diminished enhancement in apoptosis caused by RIP1 silencing, whereas genetic or pharmacological inhibition of NF-κB recapitulated the sensitizing effect of RIP1 silencing on induction of apoptosis. While RIP1 is known to be critical for activation of NF-κB in melanoma cells^13^, we and others have previously shown that NF-κB is involved in intrinsic and acquired resistance of melanoma cells to BRAF inhibitors^13, 20, 26–28^. The results from present study mechanistically established NF-κB as the effector of RIP1-mediated protection of melanoma cells from apoptosis when the RAF/MEK/ERK pathway is inhibited. NF-κB has been reported to protect melanoma cells against BRAF inhibitors through upregulation of cellular caspase-8 (FLICE)-like inhibitory protein (c-FLIP) and low-affinity nerve growth factor receptor (LNGFR; CD271)^27, 28^. Moreover, given that induction of apoptosis by inhibition of the RAF/MEK/ERK pathway involves activation of the mitochondrial apoptotic pathway^29^, it is conceivable that the anti-apoptotic Bcl-2 family members Bcl-2 and Bcl-X_L_ that are transcriptional targets of NF-κB are also involved in RIP1-mediated protection of melanoma cells from BRAF/MEK inhibitors^30, 31^.

An important finding of this study is that melanoma cells with acquired resistance to BRAF inhibitors are more critically dependent on RIP1 for survival. This was demonstrated by results showing that silencing RIP1 alone induced apoptosis in resistant melanoma cells generated by prolonged exposure to PLX4720 *in vitro* and in fresh melanoma isolates from patients post-treatment with vemurafenib. Associated with this was increased expression of RIP1 and activation of NF-κB. The latter has been preciously attributed to increased production of TNFα in the melanoma microenvironment upon treatment with BRAF inhibitors^13^, and was also related to activation of Akt in resistant melanoma cells^9^. We have previously shown that autocrine TNFα is an important element that promotes RIP1 expression and NF-κB activation in melanoma cells^13^. It therefore seems that multiple mechanisms may cooperatively contribute to the increased activation of NF-κB in melanoma cells with acquired resistance to BRAF inhibitors.

How is RIP1 upregulated in melanoma cells that have acquired resistance to BRAF inhibitors? This appeared to be due to a posttranslational increase, in that the expression levels of the RIP1 transcript remained similar in resistant melanoma cells compared with their parental counterparts. Nevertheless, the half-life time of the RIP1 protein was prolonged in resistant cells, which was associated with the decrease in K-48-linked polyubiquitination and the increase in K-63-linked polyubiquitination of the protein^13^. These results indicate that RIP1 upregulation in the cells is caused by reduction in its proteosomal degradation. Indeed, we found that downregulation of CYLD, which is one of the deubiquitinases of RIP1, was responsible for upregulation of RIP1 in melanoma cells with acquired resistance to BRAF inhibitors. CYLD is known to have a tumor suppressive role in melanoma^22^. Our results suggest that the tumor suppressive role of CYLD is, at least in part, due to its inhibitory effect on the expression of RIP1 and subsequent reduction in activation of NF-κB in melanoma cells.

**Fig. 6:**
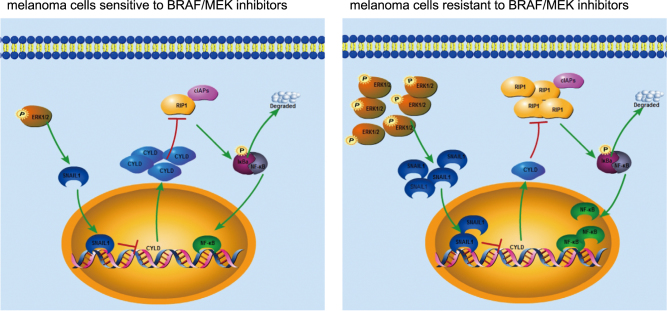
A proposed resistant mechanism to BRAF inhibitors in melanoma. A novel signaling pathway ERK-Snail1-CYLD-RIPK1-NF-κB contributes to resistance of melanoma cells to BRAF inhibitors. In brief, aberrant activation of ERK1/2 in melanoma cells resistant to BRAF inhibitors downregulates the transcription of CYLD via Snail1, which is a transcription repressor of CYLD. Consequentially, suppression of CYLD sustains cIAP-mediated K63-linked ubiquitination of RIP1, thus leading to activation of NF-κB and subsequent protection of melanoma cells from BRAF inhibitor-induced killing

In search for the mechanism(s) responsible for downregulation of CYLD in melanoma cells with acquired resistance to BRAF inhibitors, we found that Snail1, which is known to transcriptionally repress the expression of CYLD^22^, was increased in resistant melanoma cells. This was caused by the increased activation of ERK, as silencing of ERK1/2 reduced the expression of Snail1 and upregulated CYLD. Moreover, silencing of ERK1/2 caused downregulation of RIP1, which was reversed by overexpression of Snail1. These results identify the increase in ERK activation as a causal mechanism leading to upregulation of RIP1 in melanoma with acquired resistance to BRAF inhibitors. Importantly, ERK activation also appeared to play a role in maintaining RIP1 expression through upregulation of Snail1 and downregulation of CYLD in melanoma cells acquired resistance to BRAF/MEK inhibitors (Fig. 6). Therefore, RIP1 is a novel downstream effector of oncogenic activation of the BRAF/MEK/ERK pathway in melanoma.

In summary, we have found that RIP1 plays an important role in intrinsic and acquired resistance of melanoma cells to apoptosis induced by BRAF/MEK inhibitors through activation of NF-κB. Moreover, we have demonstrated that activation of the BRAF/MEK/ERK pathway is involved in regulation of RIP1 expression through promoting the expression of Snail1 and subsequent downregulation of CYLD. The practical relevance of the role of RIP1 in protecting melanoma cells from BRAF/MEK inhibitors was shown by studies in melanoma cells grown in 3D cultures, and more importantly, in paired fresh melanoma isolates before and after treatment with vemurafenib. Since RIP1-mediated NF-κB activation is mediated by its intermediate domain independently its kinase activity^32–34^, inhibitors that specifically target the intermediate domain of RIP1 may be useful in improving the therapeutic efficacy of BRAF/MEK inhibitors in the treatment of melanoma.

## Materials and methods

### Cell lines and human tissues

Human melanoma cell lines cultured in DMEM containing 5% FCS has been described previously^35^. Five paired melanoma tissue samples from patients pre- and post treatment with vemurafenib have been established and described previously^20^. Melanoma cells selected for resistance to apoptosis induced by the mutant BRAF Inhibitor PLX4720 has been described previously^9^. Individual cell line authentication has been confirmed using the AmpFISTRIdentifiler PCR Amplification Kit from Applied Biosystems (Mulgrave, VIC, Australia) and GeneMarker V1.91 software (SoftGenetics LLC, State College, PA) every 6 months. Cell lines were regularly tested for mycoplasma infection using Myco Alert according to the manufacturer’s protocol (Lonza, Walkersville, MD, USA). Studies using human tissues were approved by the Human Research Ethics Committees of the University of Newcastle and Royal Prince Alfred Hospital, Australia.

### Antibodies and reagents

The antibody against RIP1(H-207) used in immunoprecipitation and antibodies against cIAP1(F-4), cIAP2(H-85), p-ERK(E-4), JNK(D-2), p38(A-12), p-p38(E-1), and GAPDH(6C5) were purchased from Santa Cruz Biotechnology (Santa Cruz, CA). The antibodies against RIP1, phospho-IκBα, IκBα, and PARP used in western blotting were from BD Biosciences (Marrickville, NSW, Australia). Antibodies against p44/42 MAPK, K48-linkage Specific Polyubiquitin(D9D5), Phopho-SAPK/JNK(Thr183/Tyr185)(81E11), FLIP(D16A8), Phospho-NF-κB p65 (Ser536) and SNAIL(C15D3) were purchased from Cell Signaling Technology (Beverly, MA). Antibody against CYLD was purchased from Abcam(Melbourne, VIC, Australia). The antibody against K63-linked ubiquitin was from Novus Biologicals (Littleton, CO). Antibodies against caspase8 and caspase3 were from Enzo life sciences. The RIP1 kinase inhibitor necrostatin-1 (Nec-1), the Smac mimetic SM406, the caspase inhibitor z-VAD-fmk, the RAF Inhibitor Vemurafenib, the MEK inhibitor Trametinib, and the selective ERK inhibitor SCH772984 were from Selleckchem (Houston, TX). The NF-κB inhibitors PS1145 and BAY-11-7082, the Doxcyclin, the Cycloheximide(CHX), and the MG132 were from Sigma-Aldrich (Castle Hill, NSW, Australia). The NF-κB reporter assay kit was from QIAGEN (Valencia, CA). FITC Annexin V Apoptosis Detection Kit was from BD bioscience (Marrickville, NSW, Australia).

### Clonogenic assays

Cells were seeded at 1.68 × 10^5^ per well onto six-well culture plates and allowed to grow for 24 h followed by transfection. Cells were then re-seeded at 2000 per well onto six-well culture plates and allowed to grow for a further 12 days before fixation with methanol and staining with crystal violet.

### Cell viability

Cells were seeded 5000 per well onto flat-bottommed 96-well plates and allowed to grow for 24 h followed by treatment as desired. Cell Titer-Glo Luminescent Cell Viability Assay kit was used according to the manufacturer’s instructions (Promega, San Luis Obispo, CA). Luminescence was recorded by Synergy 2 multi-detection microplate reader (BioTek, VT).

### Flow cytometry and apoptosis

Immunostaining on intact and permeabilized cells was carried out as described previously^35, 36^. Analysis was carried out using a Becton Dickinson (Mountain View, CA) FACScan flow cytometer. Quantitation of apoptotic cells was carried out according to the manufacturer’s instructions of FITC Annexin V Apoptosis Detection Kit (BD Bioscience, Marrickville).

### NF-κB reporter assays

NF-κB reporter assay was performed according to manufacturer’s instructions (Qiagen)^20^. Briefly, cells transfected with the NF-κB reporter plasmids were subjected to Dual-Luciferase Reporter Assay (Promega) after desired treatment.

### Immunoblotting and Immunoprecipitation (IP)

Immunoblotting was carried out as previously described^37^. The protocol of Detection of protein ubiquitination in cultured cells is as follows:

1. Make complete cell lysis buffer (2% SDS, 150 mM NaCl, 10 mM Tris-HCL, PH8.0) with 2 mM sodium orthovanadate, 50 mM sodium fluoride, and protease inhibitors.
2. Lyse cells with 100 μl cell lysis buffer per plate (6 cm dish). Swirl the dish carefully to let the lysis buffer cover the entire area of grown cells.
3. Collect the cells with a cell scraper and transfer the cell lysates into a 1.5 ml eppendorf tube. Place the tube onto a hot plate immediately to boil for 10 mins at 95 °C.
4. Shear the cells with a sonication device.
5. Add 900 μl of dilution buffer (10 mM Tris-HCL, PH8.0, 150 mM NaCl, 2 mM EDTA, 1% Triton). Incubate samples at 4 °C for 30–60 min with rotation.
6. Spin the diluted samples at 20,000 × *g* for 30 min. Transfer the resulting supernatant to a new eppendorf tube. Be careful not to disturb the pellet.
7. Measure the protein concentration.
8. Prepare Protein A/G-agarose bead-conjugated antibody against the target protein (RIP1) in a compatible buffer (50% slurry). Cut the narrow end of a P-200 pipette tip and transfer 14–20 μl of resin to 500–1500 μg of prepared cell lysates for immunoprecipitation.
9. Incubate the cell lysate-bead mixture at 4 °C overnight with rotation.
10. Spin down the beads at 5000 × *g* for 5 min. Aspirate the supernatant. Wash the resin with the washing buffer (10 mM Tris-HCL, PH8.0, 1 M NaCl, 1 mM EDTA, 1% NP-40) twice.
11. Spin the beads for a final time at 20,000 × *g* for 30 s. Aspirate the residual washing buffer and boil the resin with 2× SDS loading buffer.
12. Load samples onto a SDS-PAGE gel for immunoblotting analysis.
13. Detect ubiquitin and the target protein with respective antibodies.

### Quantitative PCR analysis

Quantitative PCR (qPCR) was carried out as described before^37^. Briefly, the reaction was carried out for 40 cycles: 95 °C for 15 s; 60 °C for 1 min. The relative abundance of mRNA expression of a control sample was arbitrarily designated as 1. The primer sequences used are: forward, 5′-AGGCTTTGGGAAGGTGTCTC-3′, reverse, 5′-CGGAGTACTCATCTCGGCTTT-3′ for RIP1and forward, 5′-ATCTGGTGATTGAATTGGAG-3′, reverse, 5′-ATATGATAGCCCAGGGAAGT-3′ for cFLIP.

### Small interference RNA and transfection

The siRNAs were purchased from GenePharma (Shanghai, China). Transfection of siRNAs was carried out in Opti-MEM medium (Invitrogen) using Lipofectamine 2000 reagent (Invitrogen) according to the manufacturer’s transfection protocol^38^. The siRNA sequences are listed in Supplementary Table 1.

### Plasmid vectors and transfection

The lentivirus-based inducible knock-down system (FH1tUTG) was a gift from Dr. Herold M.J.^39^, DNA oligos containing the LacZ and RIP1 shRNA sequence were cloned into FH1tUTG vector. Flag-HA-CYLD (Addgene plasmid # 22544) and Flag-HA-GFP (Addgene plasmid # 22612) were purchased from Addgene^40^. RIP1 cDNA (pEZ-Lv201) and its control vector (Lv201CT) and Snail1 cDNA (pEZ-M98) and its control vector (M98CT) were from GeneCopoeia. The Primers and DNA oligo sequences of plasmids were listed in Supplementary Table 2, respectively. The plasmids were transfected as described previously^38^.

### 3D culture

3D culture was performed using the hanging drop technique as previously described^41^. Cells were stained with calcein AM (living cell stain) and ethidium homodimer-1 (dead cell stain) (Life Technologies, Scoresby, VIC, Australia) for 30 min before taken images with a fluorescence microscope(Carl Zeiss).

### Statistical analysis

Statistical analysis was performed using JMP Statistics Made VisualTM software. Student’s *t* test was used to assess differences between different groups. A *P* value <0.05 was considered statistically significant.

## Electronic supplementary material

Supplementary Figures

Supplementary Tables
