# Correction to: RIP1 protects melanoma cells from apoptosis induced by BRAF/MEK inhibitors

**DOI:** 10.1038/s41419-019-1301-2

**Published:** 2019-03-05

**Authors:** Fu Xi Lei, Lei Jin, Xiao Ying Liu, Fritz Lai, Xu Guang Yan, Margaret Farrelly, Su Tang Guo, Xin Han Zhao, Xu Dong Zhang

**Affiliations:** 1 Department of Medical Oncology, The First Affiliated Hospital of Xi’an Jiaotong University, Xi’an, Shaanxi 710061 China; 2 School of Medicine and Public Health, The University of Newcastle, Newcastle, NSW 2308 Australia; 3 School of Life Science, Anhui Medical University, Hefei, Anhui 230032 China; 4 Department of Molecular Biology, Shanxi Cancer Hospital and Institute, Taiyuan, Shanxi 030013 China

**Correction to:** **Cell Death and Disease** (2018) **9**: 679

10.1038/s41419-018-0714-7

published online 07 June 2018

Since publication of this paper, the authors have noticed that there were errors in Fig. 2A (the GAPDH of Mel-CV, Mel-CV.S, Mel-RMu and Mel-RMu.S), Fig. 2C (the GAPDH of Mel-CV.S and Mel-RMu.S), Fig. 3F (the GAPDH of Mel-CV.S and Mel-RMu.S), Fig. 3J(the GAPDH of Mel-RMu.S), Fig. 5C (the ERK1/2 of patient#3(post)), and Fig. 5F (the RIP1 of Mel-CV.S and Mel-RMu.S, the GAPDH of Mel-CV and Mel-RMu). As a result of the misfiling of the data during preparation of figures, incorrect images were inadvertently inserted in these figures. The correct figures are given below. The corrections do not alter the conclusions of the paper.

**Figure 1:**
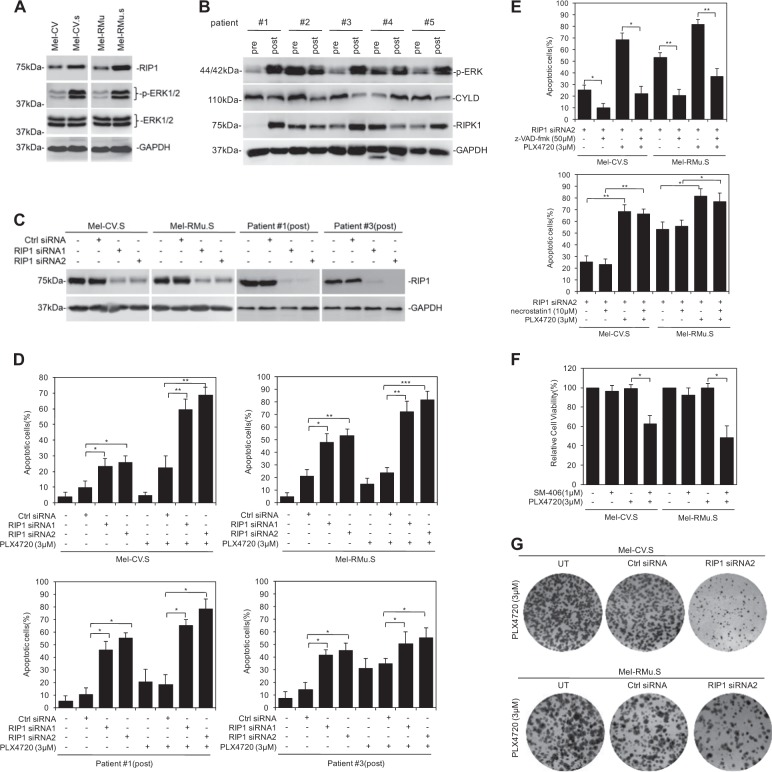
Fig. 2

**Figure 2:**
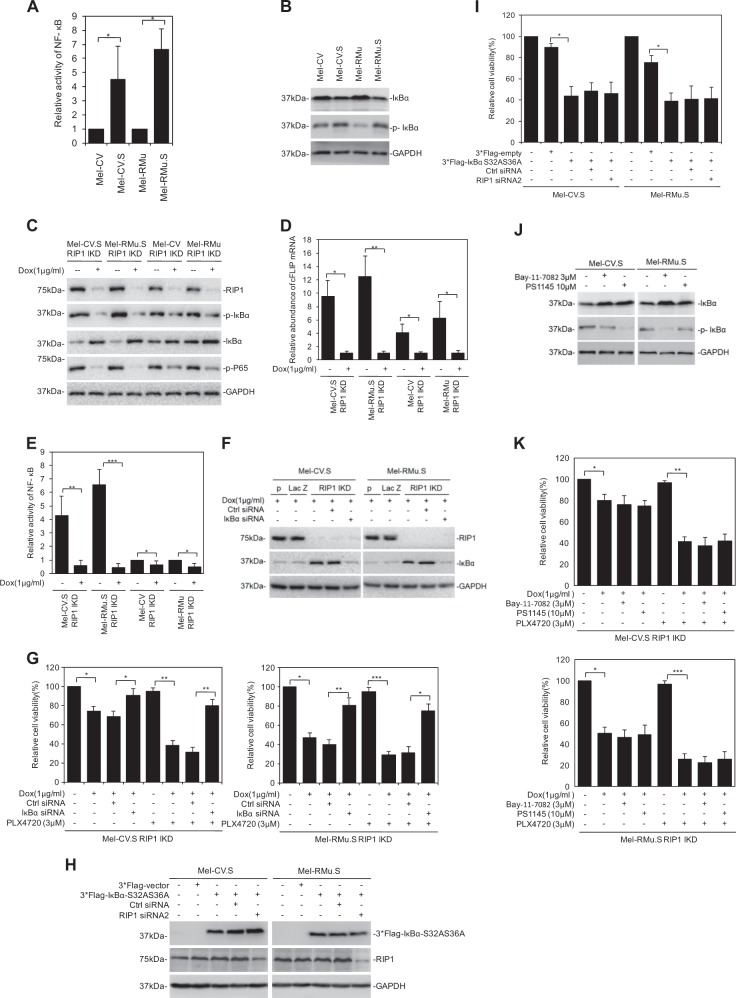
Fig. 3

**Figure 3:**
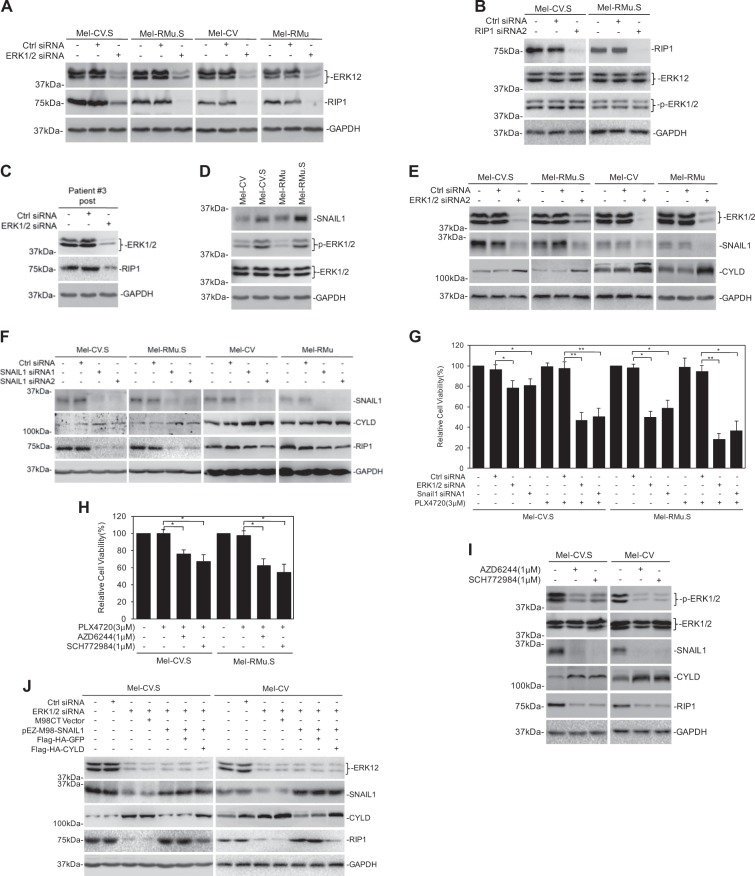
Fig. 5

The authors would like to apologize for any inconvenience this may have caused.

