# Supplementary material for: RIP1 protects melanoma cells from apoptosis induced by BRAF/MEK inhibitors
Source: Cell Death Dis. 2018 Jun 7;9(6):679. doi: 10.1038/s41419-018-0714-7 (PMC5992182; doi:10.1038/s41419-018-0714-7)
Supplement: Supplementary file 1 — Supplementary Figures [file 41419_2018_714_MOESM1_ESM.docx]

**Supplementary Fig. S1.** Cellstreated with TNFα blocking antibody in presence or absence of PLX4720 3μM were subjected to Cell Titer-Glo assays. Data are mean±SE, n=3. **P*<0.05, student’s *t* test.

a

b

**Supplementary Fig. S2. a,** whole-cell lysates were subjected to Western blotting. n=3. **b,** melanoma cells treated with Dox(1μg/ml) for 72h were subjected to Western blotting.n=3.

**Supplementary Fig. S3.** Cells individually transfected with either control siRNA or IκBα siRNA in presence of Doxycycline (1μg/ml)were subjected to Cell Titer-Glo assays. Data are mean±SE, n=3. **P*<0.05, student’s *t* test.

**Supplementary Fig. S4.** Regression analysis of the relationship between RIP1 levels and p-ERK levels as shown in Fig.2B in pairedsets of pre-treatment (pre) and post-relapsed (post) patient specimens from 5 patients with relapsed BRAF^V600E^-expressing melanomas. Data are mean±SE, n=3.
