# Supplementary material for: RIP1 protects melanoma cells from apoptosis induced by BRAF/MEK inhibitors
Source: Cell Death Dis. 2018 Jun 7;9(6):679. doi: 10.1038/s41419-018-0714-7 (PMC5992182; doi:10.1038/s41419-018-0714-7)
Supplement: Supplementary file 2 — Supplementary Tables [file 41419_2018_714_MOESM2_ESM.docx]

**Table 1: List of siRNAs**

| siRNA | Sense | Anti-sense |
| --- | --- | --- |
| ERK1 siRNA | GCUGAACUCCAAGGGCUAUTT | AUAGCCCUUGGAGUUCAGCTT |
| ERK2 siRNA | GUGCUCUGCUUAUGAUAAUTT | AUUAUCAUAAGCAGAGCACTT |
| RIP1 siRNA1 | CCUUCUGAGCAGCUUGAUUTT | AAUCAAGCUGCUCAGAAGGTT |
| RIP1 siRNA2 | GCCAGCUGCUAAGUACCAATT | UUGGUACUUAGCAGCUGGCTT |
| SNAIL1 siRNA1 | CCCACUCAGAUGUCAAGAATT | UUCUUGACAUCUGAGUGGGTT |
| SNAIL1 siRNA2 | CUCCUCUACUUCAGUCUCUTT | AGAGACUGAAGUAGAGGAGTT |
| CYLD siRNA | CAGAUUGAGCGCUGUAACUCU | AGUUACAGCGCUCAAUCUGAU |
| IκBα siRNA | CUC CGA GAC UUU CGA GGAA | UUC CUC GAA AGU CUC GGAG |
| Control siRNA | UUCUCCGAACGUGUCACGUTT | ACGUGACACGUUCGGAGAATT |

**Table 2: List of DNA oligo sequenses**

| DNA oligo | Sense | Antisense |
| --- | --- | --- |
| RIP1 | TCCCCCACTAGTCTGACGGATAATTCAAGAGATTATCCGTCAGACTAGTGG TTTTTC | TCGAGAAAAACCACTAGTCTGACGGATAATCTCTTGAATTATCCGTCAGACTAGTGG |
| LacZ | TCCCCGACTACACAAATCAGCGATTCAAGAGATCGCTGATTTGTGTAGTCGTTTTTC | TCGAGAAAAACGACTACACAAATCAGCGATCTCTTGAATCGCTGATTTGTGTAGTCG |
